# Examining the disparities of anti-malarial drug consumption among children under the age of five: a study of 5 malaria-endemic countries

**DOI:** 10.1186/s12936-023-04805-x

**Published:** 2023-12-05

**Authors:** Md Sabbir Hossain, Talha Sheikh Ahmed, Nahid Sultana, Muhammad Abdul Baker Chowdhury, Md Jamal Uddin

**Affiliations:** 1 Department of Statistics, Shahjalal University of Science and Technology, Sylhet, 3114 Bangladesh; 2 Faculty of Graduate Studies, Daffodil International University, Savar, Dhaka, 1216 Bangladesh; 3 Department of Geography and Environment, Shahjalal University of Science and Technology, Sylhet, 3114 Bangladesh

**Keywords:** Antimalarial drug consumption, Children under five, LMICs, Malaria, MIS

## Abstract

**Background:**

Malaria is one of the most prominent illnesses affecting children, ranking as one of the key development concerns for many low- and middle-income countries (LMICs). There is not much information available on the use of anti-malarial drugs in LMICs in children under five. The study aimed to investigate disparities in anti-malarial drug consumption for malaria among children under the age of five in LMICs.

**Methods:**

This study used recent available cross-sectional data from the Malaria Indicator Survey (MIS) datasets across five LMICs (Guinea, Kenya, Mali, Nigeria, and Sierra Leone), which covered a portion of sub-Saharan Africa. The study was carried out between January 2, 2023, and April 15, 2023, and included children under the age of five who had taken an anti-malarial drug for malaria 2 weeks before the survey date. The outcome variable was anti-malarial drug consumption, which was classified into two groups: those who had taken anti-malarial drugs and those who had not.

**Results:**

In the study of LMICs, 32,397 children under five were observed, and among them, 44.1% had received anti-malarial drugs. Of the five LMICs, Kenya had the lowest (9.2%) and Mali had the highest (70.5%) percentages of anti-malarial drug consumption. Children under five with malaria are more likely to receive anti-malarial drugs if they are over 1 year old, live in rural areas, have mothers with higher education levels, and come from wealthier families.

**Conclusion:**

The study emphasizes the importance of developing universal coverage strategies for anti-malarial drug consumption at both the national and local levels. The study also recommends that improving availability and access to anti-malarial drugs may be necessary, as the consumption of these drugs for treating malaria in children under the age of five is shockingly low in some LMICs.

**Supplementary Information:**

The online version contains supplementary material available at 10.1186/s12936-023-04805-x.

## Background

In 2017, there was an estimated 219 million cases of malaria, marking a 5% rise from the previous year. Moreover, over 60% of fatalities were concentrated among children under the age of five in 91 different countries [1]. Malaria is most likely to affect newborns, children under the age of five, individuals with HIV/AIDS, pregnant women, vulnerable migrants, mobile populations, and tourists. In 2020, ninety-five percent of all malaria occurrences and 96% of deaths, as well as 80% of all malaria deaths for children under five, were seen on the African continent [2]. Over the years, an unbalanced share of the global malaria burden has been seen on the African continent, which increased during the apex of the COVID-19 pandemic (2020–2021) [2]. In 2018, the countries overburdened with 228 million cases of falciparum malaria were Nigeria (25%), the Democratic Republic of the Congo (12%), and Uganda (5%) [3]. Malaria accounts for one of the major concerns of illness among children in many low and middle-income countries (LMICs). In sub-Saharan Africa, the three major causes of mortality for children under 5 years of age are malaria, pneumonia, and diarrhoea [4]. In the Asia Pacific region, *Plasmodium vivax* is the leading cause of morbidity and death in children under 5 years of age [5, 6]. Gauging the actual incidence of malaria in LMICs, including in Africa, is challenging due to the under-reporting of malaria cases and deaths [7].

The sting of infected female *Anopheles* mosquitoes, which act as carriers of *Plasmodium* parasites, is the source of malaria. Among the 5 parasite species that cause an acute febrile illness in the form of malaria, two species—*Plasmodium falciparum* and *P. vivax*—create the utmost risk, but all the species can infect children [8–10]. These two-parasite species are the most prevalent on the African continent and outside of sub-Saharan Africa, respectively.

Unconsciousness and seizure known as cerebral malaria, acute anaemia that requires urgent life-saving dissemination, heavy breathing caused by acute metabolic acidosis, jaundice, and diarrhoea are the frequent lethal symptoms for under-five children with malaria in Africa [11]. The most noticeable harmful health effect of malaria in children is anaemia. Due to the dearth of intensive care, most of these children need to depend on parenteral anti-malarial agents and supportive therapy to preclude death. According to the World Malaria Report 2022, 1 in every 10 children with critical malaria symptoms admitted to hospitals in sub-Saharan Africa dies [12, 13].

Quinoline derivatives, antifolate, and artemisinin derivatives are the three major groupings of anti-malarial medications. Artemisinin and its derivatives are advocated in combination with other anti-malarial agents owing to their short half-life and slow absorbability [14]. Currently, artemisinin-based combination therapy (ACT) is preferred to chloroquine-based therapy due to widespread chloroquine resistance [15]. Despite the substantial evidence in support of ACT and parenteral artesunate, these medications are still not being implemented all over the world and when they are used, international guidelines are not being maintained thoroughly [16, 17]. Despite the increase of chloroquine-resistant *P. falciparum* species, chloroquine did remain the main form of medication for uncomplicated *P. falciparum* malaria for a major bulk of sub-Saharan countries until after 2000 [18]. In this period, due to chloroquine resistance, mortality and morbidity increased for children under 5 years of age. In 1960, sulfadoxine-pyrimethamine (SP) was brought in as an alternative to chloroquine, however, from 2003 to 2008, it was pulled back due to diminishing effectiveness both from Southeast Asia and East Africa [18].

This study aimed to ascertain the disparities in anti-malarial drug consumption among children under the age of five in five malaria-endemic countries using a region-based geographic information system (GIS) map. Moreover, the study examined the socioeconomic factors that are associated with anti-malarial drug consumption in children under the age of five across LMICs.

## Methods

### Data source

In this cross-sectional study, five malaria-endemic countries, namely Guinea, Kenya, Mali, Nigeria, and Sierra Leone, utilizing the most recent malaria survey data available from 2020 to 2021. This survey was based on the MIS (Malaria Indicator Survey), a household survey framework developed by the Monitoring and Evaluation Working Group (MERG) as part of Roll Back Malaria, an international partnership dedicated to coordinating global malaria control efforts [19]. The MIS is made up of manuals, instructions, and questionnaires based on data from the Demographic and Health Surveys (DHS). It collects information from a representative sample of respondents on a regional and national level. The MERG Survey and Indicator Guidance Working Committee is co-chaired by the DHS Program, which has also made a substantial contribution to the MIS package's design. The MIS is conducted in almost 30 low- and middle-income countries and is frequently timed to coincide with the peak season of malaria transmission (LMICs). The survey plays a vital role in collecting essential information about the prevalence of malaria, the utilization and possession of insecticide-treated mosquito nets, and the efficacy of malaria control strategies. The Roll Back Malaria Coalition has created an MIS toolkit to assist in the execution of the survey. It also offers suggested tabulations for data analysis, instructions, questions, and manuals [19].

### Study design for MIS survey

The MIS survey employs a two-stage stratified cluster sampling technique. The initial phase was selecting certain regions or clusters. The second stage comprises methodically selecting each cluster or family within an enumeration area (EA). To facilitate cross-country comparisons, MIS surveys follow a standardized set of operating protocols encompassing sampling, questionnaire design, data collection, data cleaning, coding, and analysis.

The MIS program collects data on various key malaria indicators, including the ownership and usage of insecticide-treated mosquito nets, intermittent prenatal care, blood tests for diagnosing fever in children under the age of five, and indoor residual pesticide spraying, among other widely recognized indicators related to malaria. The MIS also collects data on home demographics and possession of things like radios, bicycles, indoor plumbing, and electricity. Anaemia and malaria parasite counts can be assessed as part of the MIS for the most vulnerable family members, such as small children and pregnant women. Participants who meet the eligibility criteria and provide their consent are requested to offer a small blood sample, typically a few drops. Trained interviewers then conduct immediate on-site testing for anaemia. The prevalence of malaria has been assessed in more than 60 DHS and MIS surveys, as well as multiple DHS surveys, and many more MIS surveys [19].

### Data harmonization

**Fig. 1:**
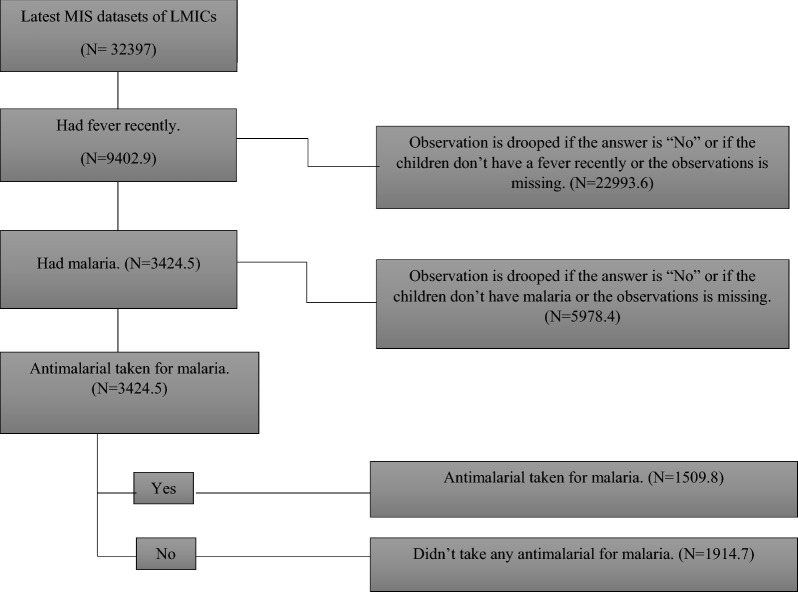
The outcome variable extraction procedure

The most recent MIS information for five LMICs was gathered from the https://dhsprogram.com/ website. To conduct the research, "Children Recode (KR)" datasets were employed, as the study exclusively focused on children under the age of five. Approximately among the 30 LMICs where MIS surveys are typically undertaken, five LIMCs were ultimately chosen (Guinea, Kenya, Mali, Nigeria, and Sierra Leone). These countries were chosen because they delivered recent MIS data and satisfied the criteria for inclusion (Fig. 1). The non-response rate for all the countries is unknown. This is often the case because, in certain countries, MIS survey reports may not be publicly released or may not be accessible in the English language.

When assessing survey datasets, it's essential to address the challenge of uneven unit selection probabilities. Sample weights are essential for generating standard errors because they help to eliminate bias that might be caused by disproportionate sampling and the consequences of non-response. As a result, leaving weights out of the analysis might produce results that are greatly biased. In the current study, sample weights were used to achieve accurate standard error and p-value calculations.

Stata introduced the term "singleton" to refer to the management of a single Primary Sampling Unit (PSU) within a stratum. Missing data is just one of several factors that can lead to a single PSU in a stratum, among other possible causes. The inability to compute standard errors is just one of several problems this creates when analysing the data. To handle single PSUs within each stratum, a scaled singleton method was employed. This involved scaling the singleton (certainty) for each stratum by computing the average of variances obtained from different sample units within those strata. Additionally, the authors ensured that categorical explanatory variable levels were accurately defined to enhance the clarity of the analysis. Following the exclusion of research variables, datasets from each country were subsequently combined.

### Data gap analysis on anti-malarial drugs consumption

The study revealed that, despite the existence of nationally representative surveys with a focus on malaria, such as the MIS, there is currently insufficient data to rate the effectiveness of the various treatment cascades for the disease. Consequently, malaria surveys may not comprehensively capture the entirety of the quality of care provided for malaria.

Second, the MIS surveys omitted information that would have been crucial for enhancing access to care for malaria, including the reasons people chose not to seek treatment. Information on blood tests was unavailable for four countries with endemic malaria. Just five of the 25 countries included in the study provided data on malaria test results that might establish the best course of therapy for febrile children under five.

Third, the data were based on mothers' accounts of the anti-malarial medications for their children received, rather than the actual prescriptions from healthcare professionals. Consequently, this might not provide an entirely accurate representation of the treatment process. Lastly, in future surveys, it could be beneficial to monitor children not only during the treatment process but also after the completion of treatment to assess the long-term impact on the quality of malaria care provided to the children. Another absence from most surveys is patient history, which should be made available because the recurrence of malaria may affect how a healthcare provider administers therapy for the disease.

Given these data gaps, the findings, which are presented in the following sections, estimate anti-malarial drug consumption patterns where data are most complete and plausible assumptions can be made to provide the essential proof of whether an anti-malarial was administered for a recent fever in each region across five LMICs.

### Explanatory variables

The MIS survey classified explanatory variables into two primary categories: the first category involves individual-level variables, while the second category pertains to community-level variables. Level one variables include the child's age, sex, the mother's highest level of education, the number of children under the age of five, and the wealth index. Level two variables cover the type of place of residence and the individual's home country. These variables were documented and then pre-coded based on the available data and the most probable causal connections. A comprehensive description of each variable can be found in Table 1.

**Table 1:** Variables recoding procedures of Malaria Indicator Survey datasets

Variables	Code at MIS Dataset	Categories in MIS	Recoding procedure
Level one variables (individual level variables)			
Number of children under 5	v137	One	Recoded as 1 = "one", 2 = "two", 3 = "three ", 4 = "four", 5 = "five or above"
		Two	
		Three	
		Four	
		Five or above	
Age of the child (in years)	hw1	One year old	Recoded as 1 = “min/12 months”, 2 = “13/24 months”, 3 = “25/36 months”, 4 = “37/48 months”, and 5 = ”49/60 months”
		Two years old	
		Three years old	
		Four years old	
		Five years old	
Sex of the child	b4	Male	Recoded as 1 = ” male” and 2 = “female”
		female	
Wealth index	v190	Poorest	Recoded as 1 = “poorest”, 2 = “poorer”, 3 = “middle”, 4 = “richer” and 5 = “richest”
		Poorer	
		Middle	
		Richer	
		Richest	
Had fever in last two weeks	h22	Yes	Recoded as 1 = “Yes” 0 = “No”
		No	
Had malaria	S406	Yes	Recoded as 1 = “Yes” 0 = “No”
		No	
Mother’s highest level of education	v106	No education	Recoded as 0 = “no education”, 1 = “primary”, 2 = “secondary”, and 3 = “Higher”
		Primary	
		Secondary	
		Higher	
		No education	
		Yes	
Level two variables (community variables)			
Type of place of residence	v025	Urban	Recoded as 1 = “Urban” 2 = “Rural”
		Rural	
Country code	v000	This cell needs a hand	
Dependent variables			
Anti-malarial taken for malaria	This was deducted by code from the dataset	Yes	Recoded as 1 = “Yes” 0 = “No”
		No	
Random effect variables			
Sampling weight	v005	For weighting the observation	
Primary sampling unit (PSU)	v021	For sample selection	
Strata	v022	For stratification	

### Outcome variable

The outcome variable, labelled "Anti-malarial Drug," is a binary variable with categories "Yes = 1/No = 0". Initially, inquiries were made regarding recent experiences of fever among the children. If the response was affirmative, subsequent questions were posed to determine whether they had received anti-malarial medication for the treatment of the fever (Fig. 1).

### Statistical analysis

Several descriptive statistics on anti-malarial exposures in relation to the explanatory variables and the countries were computed. All the findings that are shown in the tables and figures are based on weighted estimations. Geospatial analysis was performed to give a complete picture of the anti-malarial exposures in each region of the different LMICs.

In pursuit of a meaningful association between the variables of interest and anti-malarial exposures, univariate analysis was performed on the pooled data. Using a univariate logistic regression model, it was determined if anti-malarial exposures and specific explanatory factors had any meaningful relationships. Also, an assessment was conducted on the impact of explanatory variables on anti-malarial drug consumption administered to children under five with malaria using a final multivariable logistic regression on the pooled data. The logistic regression model then includes the variables found in the univariate analysis (p-value < 0.5) [20]. A backward selection procedure, the Akaike Information Criterion (AIC), and the ROC curve were employed to get the best model. Moreover, multicollinearities for all explanatory variables in the final multivariable logistic regression was examined using the variance inflation factor (VIF). A widely accepted guideline suggests that a VIF value exceeding 10 may indicate problematic multicollinearity. No evidence of collinearity among the explanatory variables was found (mean VIF = 1.3, min VIF = 1.0, max VIF = 1.7).

This study adhered to the Strengthening the Reporting of Observational Studies in Epidemiology (STROBE) Statement—a checklist of items that should be included in the reporting of cross-sectional studies (Additional file 1: Checklist). Stata 14 version [21] and ArcGIS 10.2.7 version [22] were used for all statistical analyses.

## Results

### Descriptive analysis

Throughout the result section, weighted frequency and row percentage were utilized in the descriptive analysis. Sampling weights were implemented in all analyses to ensure a proper estimate of the p-value and confidence interval, which represent the whole population.

After pooling the individual country MIS datasets of five LMICs, a total of 32,397 children under the age of five were identified. Among them, 51.2% are males, and 48.8% are females. Furthermore, it was observed that 9402 children under the age of five had experienced a fever 2 weeks before the survey days; however, not all of them had malaria. 3424 of these children were found to have malaria. Finally, 1509 (44.1%) children under the age of five had received anti-malarial drugs (Fig. 1).

### Country descriptive

**Fig. 2:**
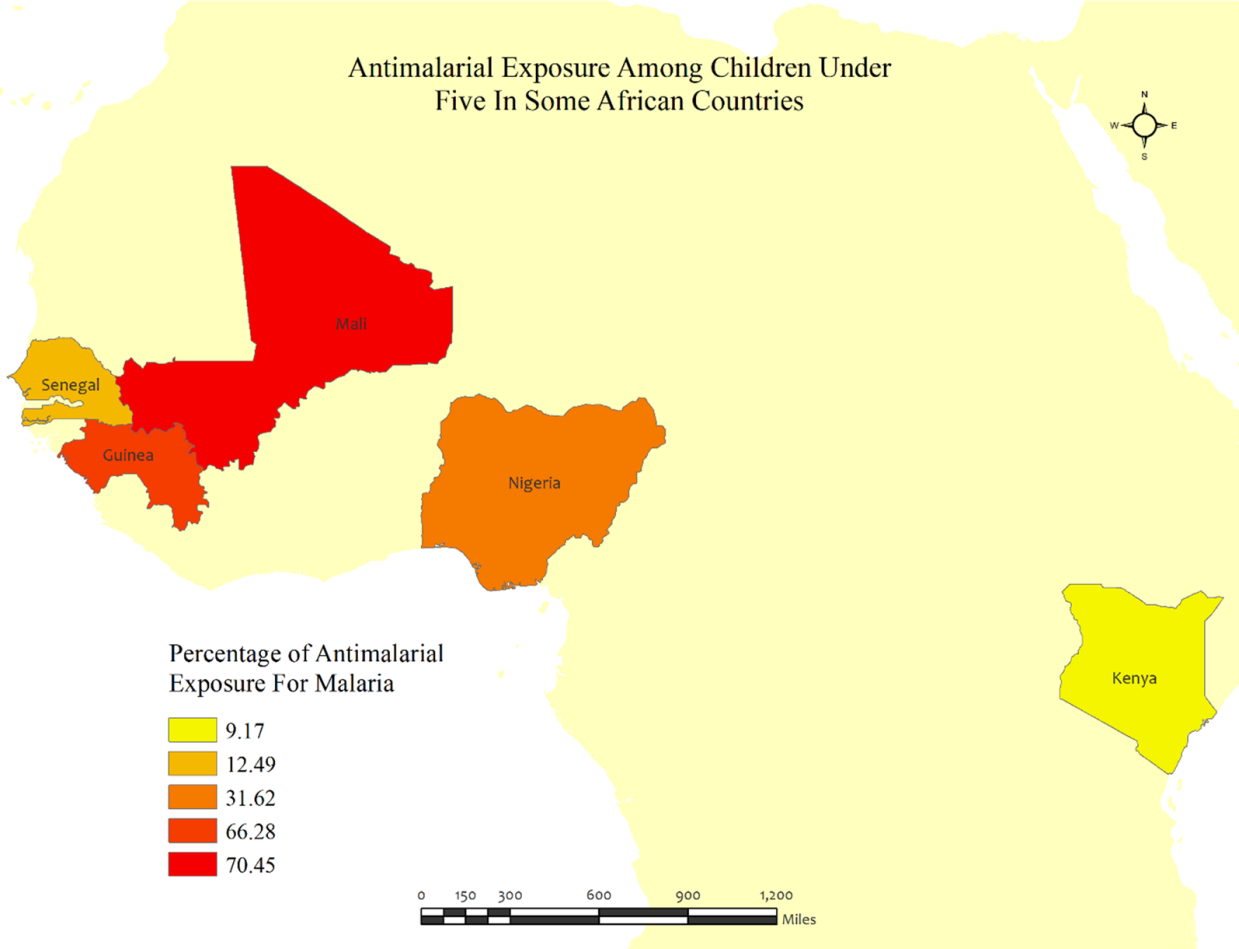
The overall prevalence of anti-malarial exposures for malaria in children under five across the five LMICs (Guinea, Kenya, Mali, Nigeria, and Senegal). The darker shades of red indicate high anti-malarial drug consumption

Kenya has the lowest (9.2%) percentage of anti-malarial consumption in children under five among the five LMICs, whereas Mali has the highest (70.5%). Guinea, Nigeria, and Senegal had anti-malarial consumption of 66.3%, 31.6%, and 12.5%, respectively. Senegal has the lowest (12.9%) percentage of malaria, and Nigeria has the highest (46.1%). Children who recently experienced a fever have a high prevalence of malaria, except for those in Senegal (Table 2, Fig. 2).

**Table 2:** Descriptive statistics of anti-malarial drugs use in children under five for malaria in 5 LMICs

Country	Had fever recently, weighted N (%)	Had malaria, weighted N (%)	Anti-malarial taken for malaria, weighted N (%)
Guinea	909.9 (23.3)	353.2 (39.2)	234.1 (66.3)
Kenya	559.2 (17.4)	127.4 (23.0)	11.68 (9.2)
Mali	2494 (27.3)	960.5 (38.7)	676.6 (70.5)
Nigeria	3917 (36.8)	1776 (46.1)	561.4 (31.6)
Senegal	1654 (30.0)	207.8 (12.9)	25.95 (12.5)

### Region-wise descriptive of anti-malarial drug consumption in five LMICs

**Fig. 3:**
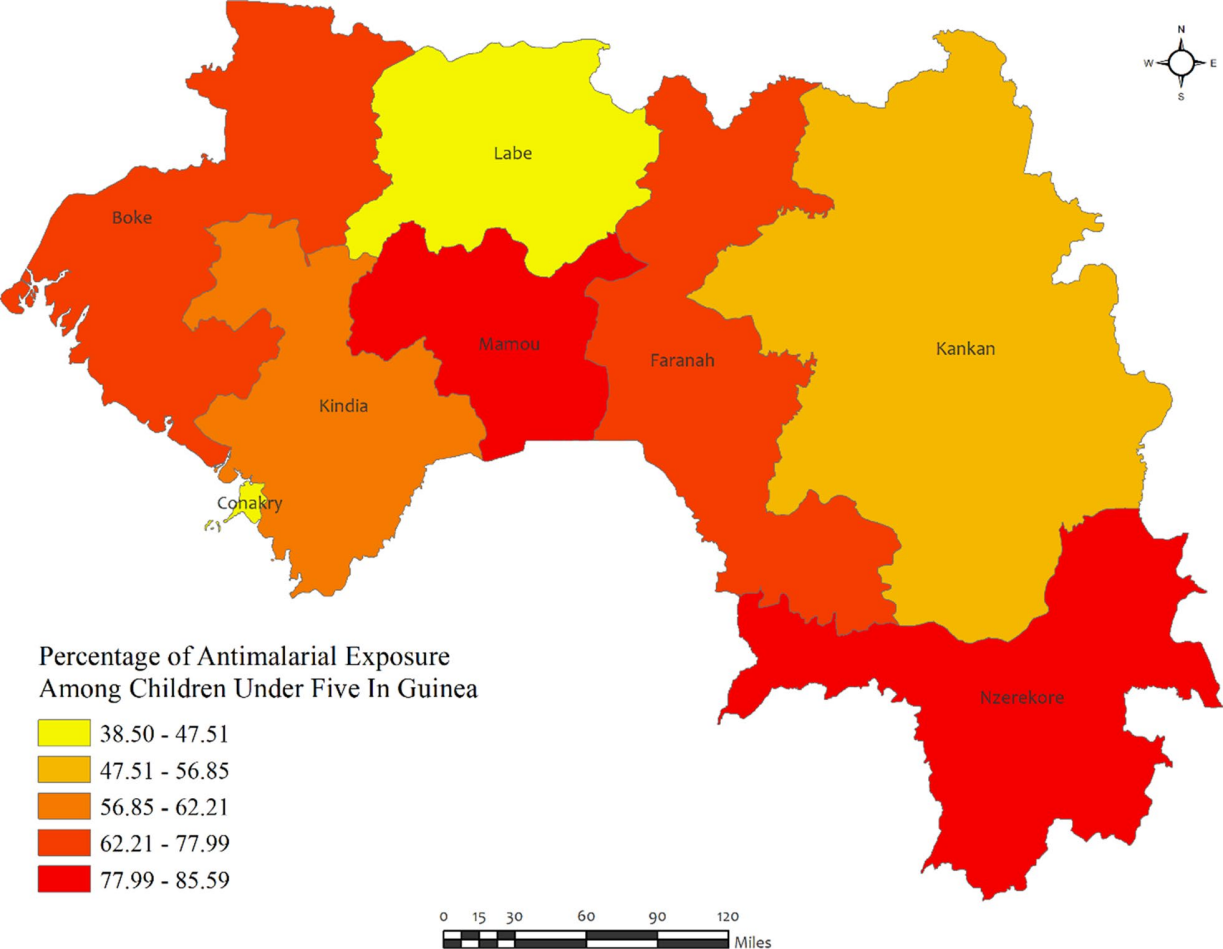
Region wise prevalence of anti-malarial exposures for malaria in children under five in Guinea. The darker shades of red indicate high anti-malarial drug consumption

Figure 3 shows that in Guinea, the overall percentages of anti-malarial consumption in children under five were high in all regions, with the highest percentages observed in Mamou (85.6%) and Nzerekore (82.6%) regions, while the lowest percentages were observed in Conakry (38.5%) and Labe (47.5%) regions (Fig. 3). However, the prevalence of malaria was quite high (above 30%) in all the regions of Guinea, except for Labe (21.7%) (Additional file 2: Table S1)**.**

**Fig. 4:**
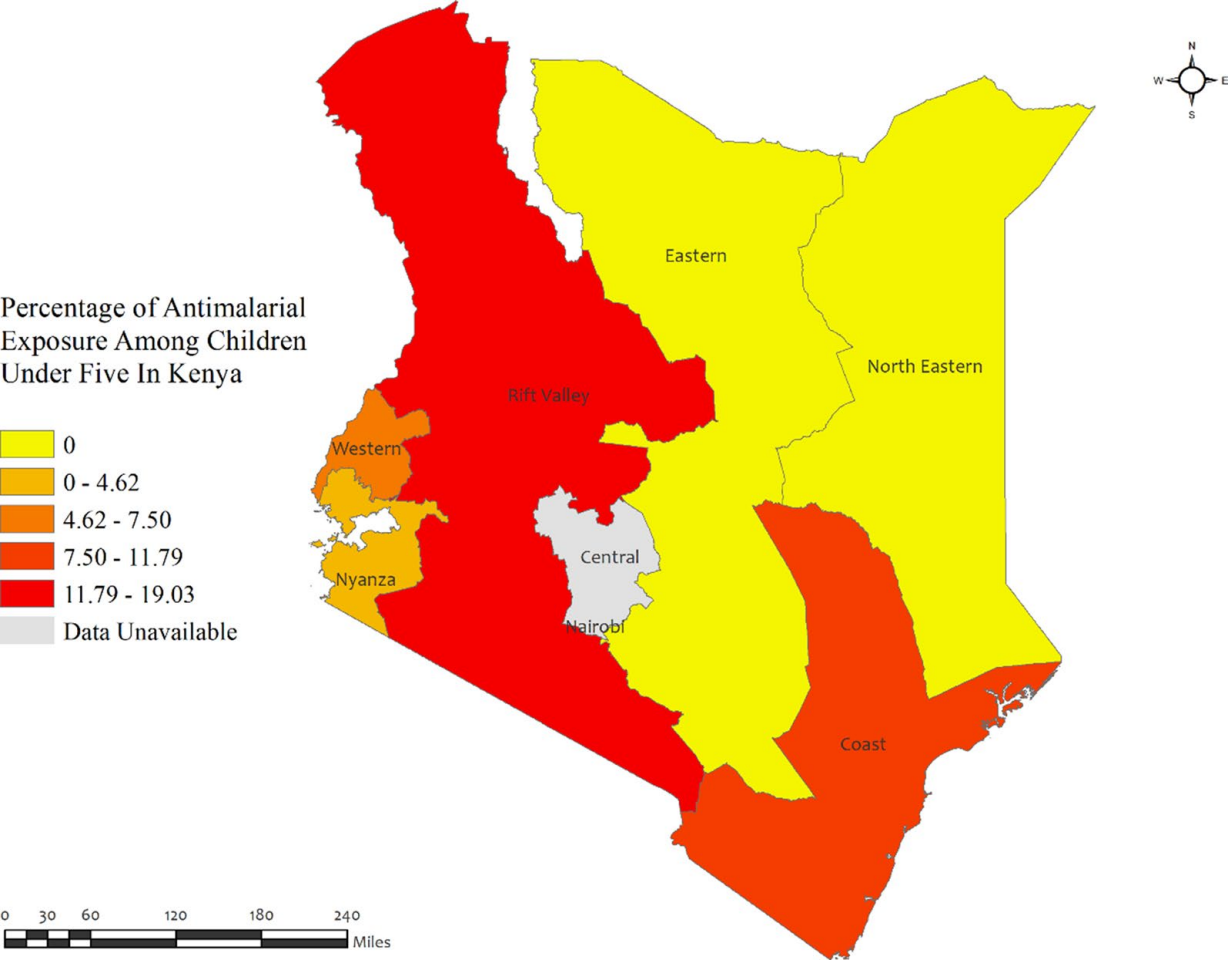
Region wise prevalence of anti-malarial exposures for malaria in children under five in Kenya. The darker shades of red indicate high anti-malarial drug consumption

In Kenya, the percentage of anti-malarial consumption in children under five was very low, with the highest percentages observed in the Rift Valley (19.0%) region and the lowest observed in the North-Eastern and Eastern regions (approximately zero percent) (Fig. 4). However, the prevalence of malaria was highest in the Nyanza (42.8%) and Western (33.3%) regions, while it was lowest in the Central and Nairobi regions (approximately zero percent) (Additional file 2: Table S2).

**Fig. 5:**
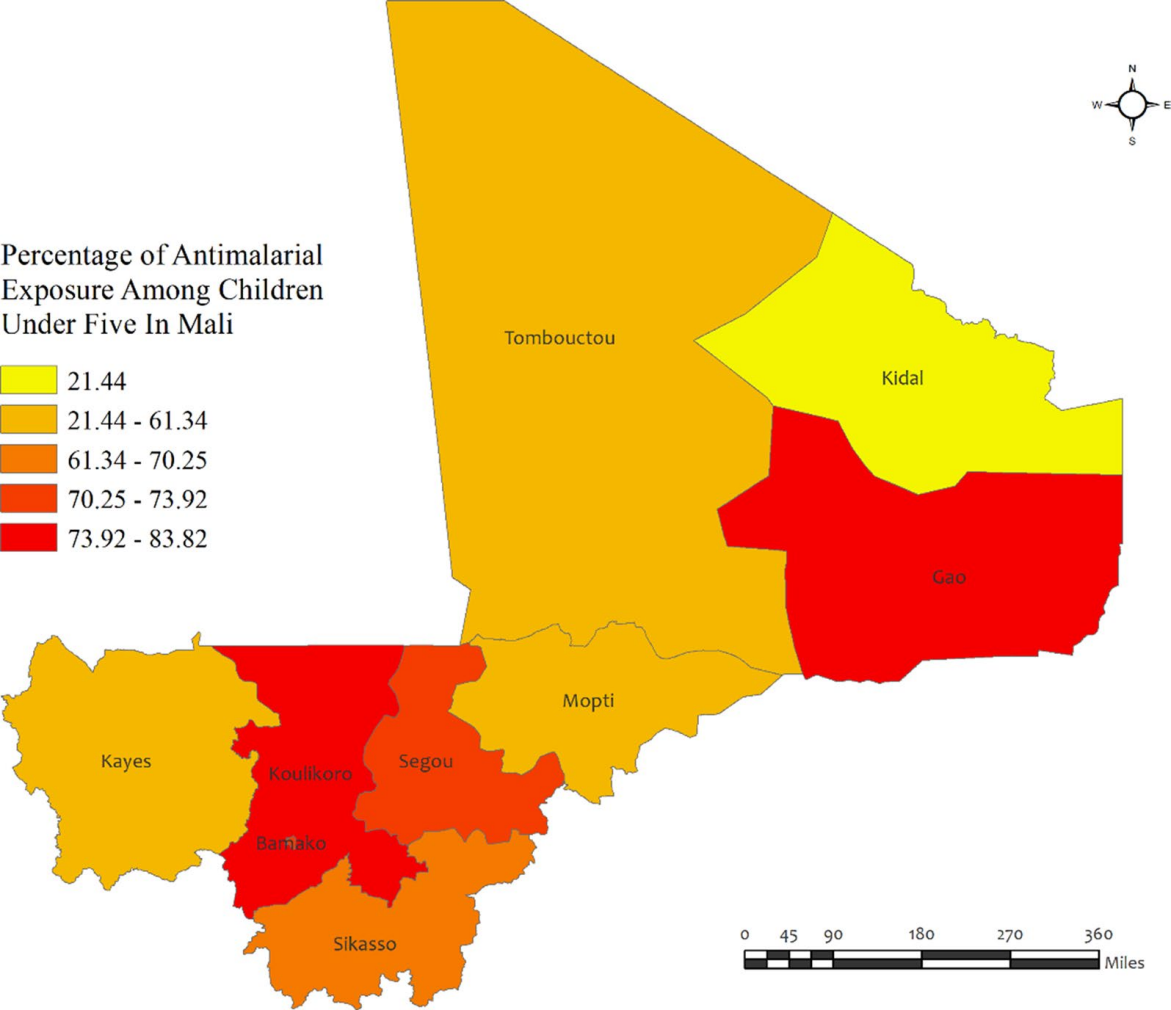
Region wise prevalence of anti-malarial exposures for malaria in children under five in Mali. The darker shades of red indicate high anti-malarial drug consumption

In Mali, the percentage of anti-malarial consumption in children under five was very high (above 60%) in all regions, except for Kidal (21.44%). The highest percentages of anti-malarial drug consumption were observed in the Koulikor (83.8%) and Gao (81.4%) regions, while the lowest was observed in Kidal (21.44%). The prevalence of malaria was quite high (above 30%) in all regions of Mali, except for Bamako (20.4%). However, the prevalence of malaria varied significantly across different regions of the country (Fig. 5). The highest percentages of malaria cases were observed in Tombouctou (51.0%) and Segou (48.1%), while the lowest incidence was recorded in Bamako (20.4%) (Additional file 2: Table S3).

**Fig. 6:**
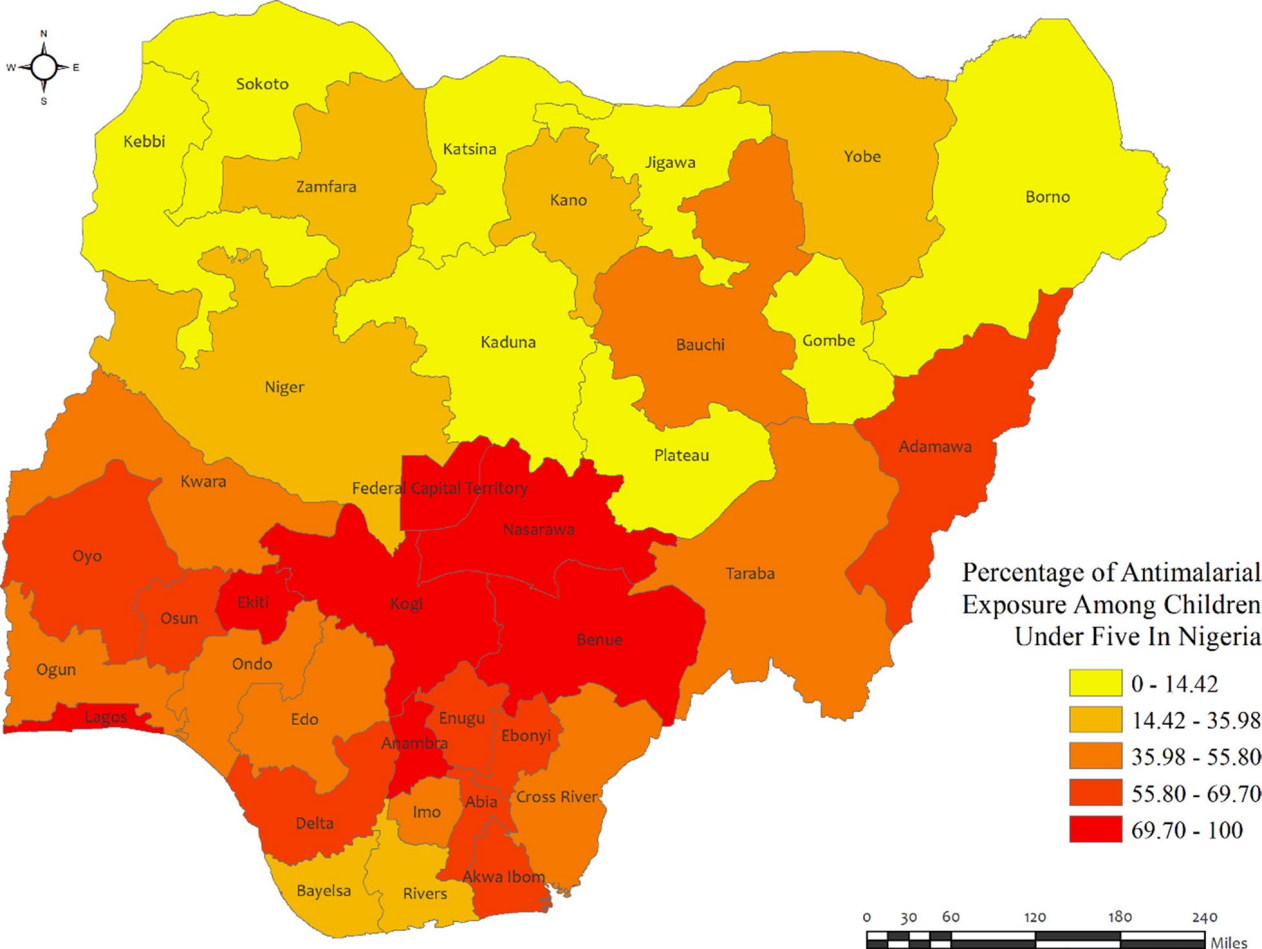
Region wise prevalence of anti-malarial exposures for malaria in children under five in Nigeria. The darker shades of red indicate high anti-malarial drug consumption

In Nigeria, the percentages of anti-malarial drug consumption in children under five were very high in most regions, with the highest percentages observed in Ekiti (100%), Kogi (85.8%), and Benue (83.8%) regions, while the lowest was observed in the Borno region (approximately zero percent)** (**Fig. 6). Most regions in Nigeria exhibit a high prevalence of malaria, with the highest percentages observed among the population in the Anambra (74.1%) and Benue (63.8%) regions, while the lowest was observed in the Akwa Ibom (12.2%) regions (Additional file 2: Table S4).

**Fig. 7:**
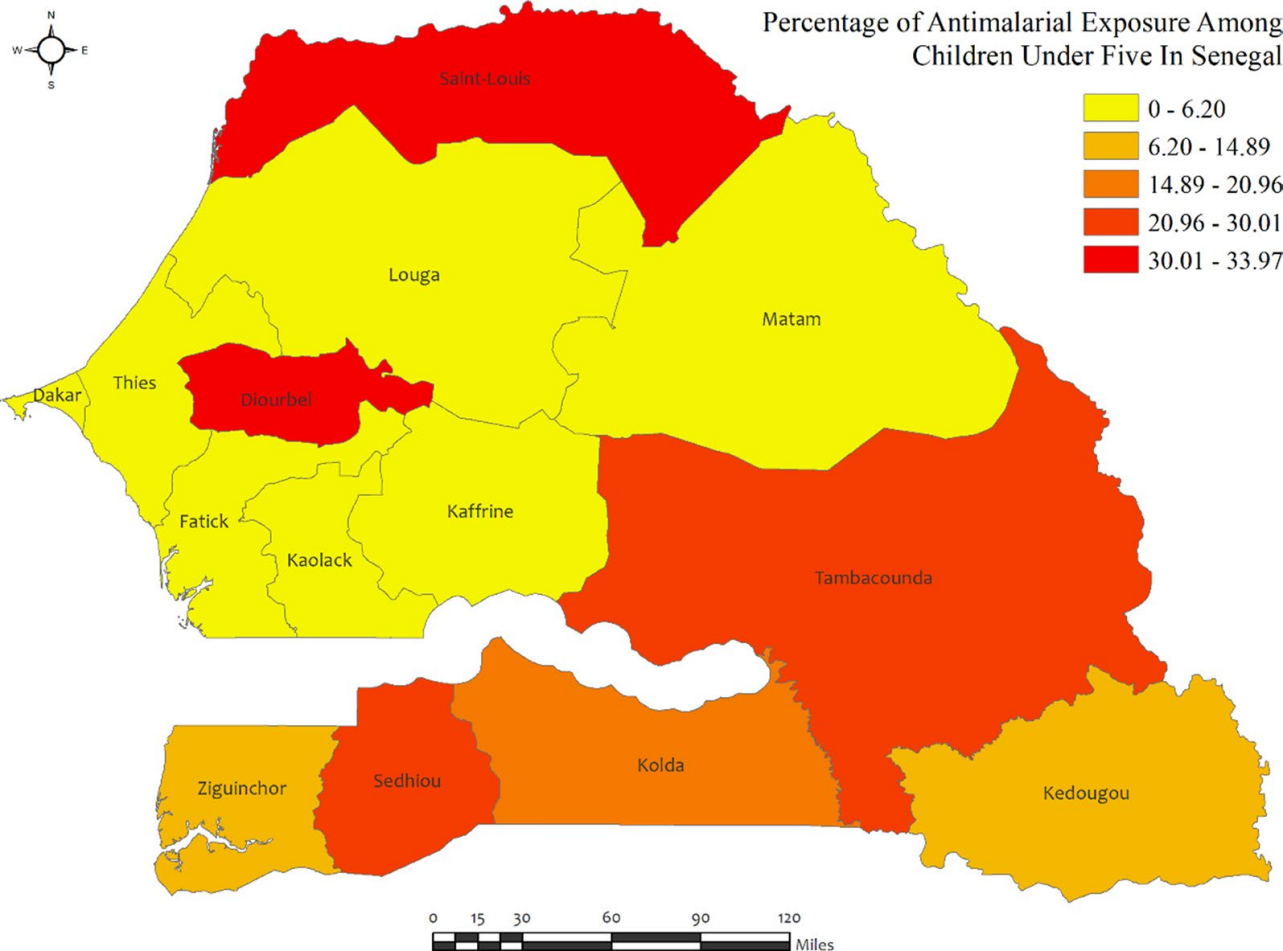
Region wise prevalence of anti-malarial exposures for malaria in children under five in Senegal. The darker shades of red indicate high anti-malarial drug consumption

In Senegal, the percentages of anti-malarial drug consumption in children under five were very low in most regions, with the highest percentages observed in Saint Louis (34.0%) and Sedhiou (30.0%) regions, while the lowest was observed in Kaffrine, Fatick, Louga, Thies, and Kaolack regions (approximately zero percent) (Fig. 7). Most regions in Senegal report a low prevalence of malaria, indicating a minimal burden of the disease among the population, with the highest percentage observed in Matam (58.8%) and the lowest observed in Louga (2.4%) (Additional file 2: Table S5).

### Descriptive statistics for explanatory variables

Table 3 shows the descriptive statistics for several explanatory factors and anti-malarial drug consumption for malaria in children under the age of five in the pooled data. In Table 3, the weighted row percentage was used.

**Table 3:** Descriptive statistics of socio-economic variables associated with anti-malarial drugs use for malaria in the pooled data

Variables	Category	Anti-malarial taken for malaria, weighted N (%)	Missing percentages (%)
Child’s age	One year old	195 (37.5)	6.2
	Two years old	322.8 (42.9)	
	Three years old	377.8 (50.9)	
	Four years old	315.3 (51.3)	
	Five years old	268.7 (46.1)	
Sex of the child	Male	820.9 (44.3)	0
	Female	688.9 (43.8)	
Type of place of residence	Urban	335.1 (46.0)	0
	Rural	1175 (43.6)	
Highest educational level of mother’s	No education	767.2 (40.2)	0
	Primary	258.4 (39.2)	
	Secondary	380.7 (54.8)	
	Higher	103.4 (63.9)	
Number of children under 5 in the household	One	327.9 (45.3)	0
	Two	448.8 (42.0)	
	Three	286.8 (42.0)	
	Four	158.9 (42.5)	
	Five or above	287.4 (50.1)	
Wealth index combined	Poorest	261.5 (33.4)	0
	Poorer	299.1 (36.1)	
	Middle	350.8 (45.4)	
	Richer	312 (54.5)	
	Richest	286.3 (61.0)	

About 44.3% of children who are male have taken anti-malarial drugs, whereas 43.8% of children who are female have taken anti-malarial drugs. The percentage of anti-malarial consumption seems to increase as the age of children increases (1 year; 37.5%, 2 years; 42.9%, 3 years; 50.9%; 4 years; 51.3%), except for 5 years with 46.1%. Children who live in urban areas received 46.0% of the anti-malarial drugs, compared to 43.6% in rural areas. Among the children, 63.9% of those whose parents had higher education took anti-malarial drugs, while only 40.2% of children whose parents had no education received such treatment. The percentage of anti-malarial drug usage varied with the number of children in the household, as follows: one child, 45.3%; two children, 42.0%; three children, 42.0%; four children, 42.5%; and five children, 50.1%. Furthermore, there appears to be an increasing trend in the percentage of children taking anti-malarials based on the wealth index of the family, with the percentages rising in ascending order from the poorest (33.4%), poorer (36.1%), middle (45.4%), richer (54.5%), to the richest (61.0%) (Table 3).

### Univariate logistic regression models in the pooled data

Table 4 illustrates the univariate relationship between specific explanatory variables and the consumption of anti-malarial drugs in children under the age of five. To ensure accurate standard error and p-value estimates in the univariate analysis, proper sampling weights were applied. Three explanatory variables were identified in the analysis as statistically significant at a 5% level of significance. The significant explanatory variables are the child’s age, the highest educational level of the mother, and the wealth index. The rest of the variables were found to be insignificant (Table 4).

**Table 4:** Univariate analysis of socio-economic variables associated with anti-malarial drugs use for malaria in the pooled data

Variables	Category	Odds ratio (OR)	P-value	Confidence interval (CI)
Child’s age	One year old	Ref		
	Two years old	1.3	0.12	[0.9–1.7]
	Three years old	1.7	< 0.01	[1.3–2.3]
	Four years old	1.8	< 0.01	[1.3–2.3]
	Five years old	1.4	0.02	[1.0–1.9]
Sex of the child	Male	Ref		
	Female	1.0	0.82	[0.8–1.2]
Type of place of residence	Urban	Ref		
	Rural	0.9	0.46	[0.7–1.2]
Highest educational level of mother’s	No education	Ref		
	Primary	1.0	0.76	[0.7–1.3]
	Secondary	1.8	< 0.01	[1.4–2.4]
	Higher	2.6	< 0.01	[1.8–3.9]
Number of children under 5 in the household	One	Ref		
	Two	0.9	0.31	[0.7–1.1]
	Three	0.9	0.38	[0.6–1.1]
	Four	0.9	0.58	[0.6–1.3]
	Five or above	1.2	0.24	[0.9–1.7]
Wealth index combined	Poorest	Ref		
	Poorer	1.1	0.55	[0.8–1.7]
	Middle	1.7	0.01	[1.1–2.5]
	Richer	2.4	< 0.01	[1.6–3.5]
	Richest	3.1	< 0.01	[2.1–4.6]

### Final multivariable logistic regression model in the pooled data

Table 5 presents the relationship between chosen explanatory variables and the consumption of anti-malarial drugs in children under the age of five. Proper sampling weights were applied to ensure an accurate standard error and p-value estimate in the univariate analysis. In the final binary multivariable logistic regression model, the variables found to be significant in the univariate analysis (p-value < 0.5) were included. The odds of receiving the anti-malarial drugs in children under five were more likely in rural areas (OR = 1.4, 95% CI 1.2–1.7) as compared to urban areas. Children whose mothers had primary (OR = 1.3, 95% CI 1.1–1.5), secondary (OR = 1.8, 95% CI 1.5–2.1), or higher education (OR = 2.7, 95% CI 2.1–2.1–3.5) education were more likely to receive the anti-malarial drug in children under five than those with illiterate mothers. The odds of receiving the anti-malarial drugs were more likely for children aged 2 years (OR = 1.6, 95% CI 1.4–1.9), 3 years (OR = 2.0, 95% CI 1.7–2.4), 4 years (OR = 2.1, 95% CI 1.8–2.6), and five (OR = 1.9, 95% CI 1.6–2.3) compared to 1 year. Children whose wealth index was poorer (OR = 1.0, 95% CI 0.9–1.2) are equally likely, middle (OR = 1.5, 95% CI 1.3–1.8), richer (OR = 1.4, 95% CI 1.2–1.7), and richest (OR = 1.7, 95% CI 1.4–2.2) were more likely to receive the anti-malarial drug in children under five than those with the poorest index. The final logistic model's ROC curve was 0.68, which indicates that the model is well-fitted (Table 5).

**Table 5:** Final binary logistic regression model adjusted with country variations in the pooled data

Variables	Category	Odds ratio	P-value	Confidence interval
Child’s age	One year old	Ref		
	Two years old	1.6	< 0.01	[1.4–1.9]
	Three years old	2.0	< 0.01	[1.7–2.4]
	Four years old	2.1	< 0.01	[1.8–2.6]
	Five years old	1.9	< 0.01	[1.6–2.3]
Type of place of residence	Urban	Ref		
	Rural	1.4	< 0.01	[1.2–1.7]
Highest educational level	No education	Ref		
	Primary	1.3	< 0.01	[1.1–1.5]
	Secondary	1.8	< 0.01	[1.5–2.1]
	Higher	2.7	< 0.01	[2.1–3.5]
Wealth index combined	Poorest	Ref		
	Poorer	1.0	0.7	[0.9–1.2]
	Middle	1.5	< 0.01	[1.3–1.8]
	Richer	1.4	< 0.01	[1.2–1.7]
	Richest	1.7	< 0.01	[1.4–2.2]

## Discussion

Malaria is one of the main causes of illness-related burden among children and one of the biggest developments and health issues for many LMICs [23]. The disparities of anti-malarial consumption among children under the age of five in LMICs were ascertained in order to address the information gap about anti-malarial drug consumption. According to the findings, the percentage of anti-malarial drug consumption in children under five was quite moderate (44.1%) across the five LMICs. However, the percentages of anti-malarial drug consumption for malaria in Mali and Nigeria were very high (over 65 percent). Conversely, Kenya and Senegal had less than 13 percent anti-malarial exposure rates.

There are several reasons for low anti-malarial exposures in some LMICs. According to a study conducted in Kenya, the biggest obstacles to successful malaria treatment are the cost of therapy, the seasonality of the sickness, financial sources, payments made informally or "under the table", relationships between providers and patients, attitudes of health professionals perceptions of the origins of sickness, the success of therapy, mistrust in the standard of care, poor compliance with therapy opening times for the facility, a lack of drugs, and where the medical facilities located [24]. Moreover, in countries where malaria is endemic, using traditional and herbal medicines appears to be the alternative treatment of choice rather than using anti-malarial medicine [25]. According to the WHO, 80% of people in the Third World depend on herbal treatments [26]. Sixty percent of people in Kenya treated their own cases of malaria at home with herbal medications obtained from local markets [27]. Similarly, a study conducted in Senegal found that the well-known trend of self-medication and an easily available alternative medicine are also responsible for the decline in the usage of anti-malarial medications [28]. Therefore, traditional, herbal, and self-medications are the major concerns for low anti-malarial exposures in malaria-endemic countries [29, 30].

Inadequate healthcare infrastructure is responsible for the country's low anti-malarial exposure rates. The healthcare system has several difficulties, such as inadequate healthcare facilities and insufficient funding to meet healthcare requirements. As a result, people who require anti-malarial drugs are less likely to get access to them. Reduced anti-malarial exposures are also a result of limited healthcare utilization. Many individuals wait until they are gravely ill before seeking medical attention. Delaying treatment increases the risk of developing severe malaria and other consequences, which makes the condition more challenging to cure [31–33].

Anti-malarial consumption for malaria differs among the LMICs. As an example, higher anti-malarial exposures were identified in Guinea and Mali compared to other LMICs. According to the research findings, one of the reasons is that malaria prevention rates in such countries are relatively high (over 35 percent). They have limited access to reliable malaria diagnosis and treatment, which is another factor. Artemisinin-based combination therapy (ACT), the usual treatment for uncomplicated malaria, is very efficient in curing the disease. Consequently, those patients have repeatedly taken anti-malarials for recent fever since they have limited access to ACT due to weak healthcare facilities and resources [34, 35]. The likelihood of children under the age of five consuming anti-malarial drugs for malaria can vary based on various socioeconomic factors.

Children in rural areas typically receive more anti-malarials for malaria than those in urban areas. There are several reasons for the high anti-malarial consumption in rural areas. The World Health Organization has reported that the species of *Anopheles* mosquito, responsible for transmitting the malaria parasite, are widespread in rural regions of sub-Saharan Africa, where many malaria cases are recorded. In addition to having restricted access to preventative measures like insecticide-treated bed nets and indoor residual spraying, rural regions often have worse housing conditions, which might make them more attractive mosquito breeding grounds. Furthermore, compared to urban areas, rural communities frequently have less developed healthcare infrastructure and resources. As a result, it could be more difficult for people in rural areas to receive an appropriate diagnosis and a malaria treatment that works. Community health workers or other non-medical employees may be the primary point of contact for people looking for healthcare services in many rural locations. Because of the lack of diagnostic resources and the need to administer rapid treatment for malaria, these individuals may be more inclined to offer anti-malarial medication to people presenting with malaria symptoms. Finally, due to their livelihoods and everyday activities, those who reside in rural regions may be more exposed to malaria. Rural dwellers could be more inclined to work outside or spend more time outside, which would increase their exposure to mosquito bites and malaria risk. The incidence of malaria may rise because of this increased exposure, increasing the requirement for anti-malarial medication [36–39].

Mothers' education was found to have a significant impact on their children’s anti-malarial consumption. Children whose mothers had higher education tend to receive more anti-malarial drugs for malaria than children whose mothers had no formal education. This is not surprising, as educated people tend to have better health-seeking abilities. They tend to go themselves and take their children to healthcare facilities more often than less educated ones [37, 40]. Education among caregivers is associated with effective anti-malarial care [41]. Children in Zambia who had caregivers with some educations were less likely to misuse anti-malarial drugs [42]. Education may influence patients' ability to understand clinic instructions, the quality of their relationship with their healthcare provider, and their ability to understand visual instructions, according to a study conducted in Uganda [43]. Along with education, the wealth index also influences the rate of anti-malarial exposure to malaria in children under five. Children in richer households are more likely to receive anti-malarial treatment than those in poorer households. The reason is like the case of education. Educated people visit hospitals or healthcare facilities far more often than poorer ones.

Children's age was found to have a significant impact on their anti-malarial consumption for malaria. Compared to newborns under 1 year old, children between the ages of 2 and 5 are more likely to get anti-malarial medications due to a higher risk of having severe malaria. Because their immune systems are still growing, children in this age range are less able to fend off the malaria parasite. The natural immunity to malaria that older children and adults have established from repeated exposure to the disease may not have been formed in young children in this age range. Moreover, antibodies developed from mothers during pregnancy still frequently provide protection for infants under the age of one. When a child gets older and the antibodies start to wear off, this protection may diminish, leaving them more vulnerable to malaria infection. Hence, unless they are showing signs of malaria, young children who have not yet lost this protection might not need anti-malarial medication [2, 44, 45].

## Recommendations

According to this study, several LMICs (Kenya, Senegal, and Nigeria) have very poor anti-malarial drug consumption for children under the age of five who have malaria. Anti-malarial drugs should be accessible and available at all levels of the healthcare system, according to nations. Effective supply chain management is required for this, including forecasting, purchasing, and distribution. To support vulnerable groups, such as children aged 2 to 5 and those with illiterate mothers, it is essential to implement targeted interventions. Possible interventions encompass health education programs, the provision of healthcare services without cost, and improving accessibility to ACT. Furthermore, raising public awareness about appropriate anti-malarial drugs can be accomplished through community outreach efforts and public health campaigns.

## Strength

This study represents a pioneering endeavour in investigating anti-malarial drug consumption in children under the age of five. Advanced geospatial analysis was utilized to develop a detailed and comprehensive portrayal of anti-malarial drug consumption in each of the five Low- and Middle-Income Countries (LMICs) where endemic malaria is prevalent, and data on malaria test results were available. This innovative approach provides a valuable resource, allowing local and national policymakers to gain timely access to a comprehensive perspective on anti-malarial drug consumption through region-specific GIS maps.

## Limitations

This study has several limitations. First, more country studies would be preferable, but only five countries malaria test results are available. Second, specific MIS datasets contained incredibly few observations, which produced some surprising findings. Finally, the study only looked at the anti-malarial drugs taken for malaria in children under five. Further research is required for all age groups.

## Conclusion

The study reveals that while some Low- and Middle-Income Countries (LMICs) exhibit a high prevalence of anti-malarial drug usage for malaria treatment (such as Mali and Guinea), others receive lower dosages (Kenya, Senegal, and Nigeria). Anti-malarial drug consumption in children under five is influenced by various factors, including the type of residence, the child's age, the mother's highest level of education, and the wealth index. The analysis recommends putting in place different anti-malarial medicine checks and balances as well as a more robust health infrastructure. It is also recommended to alert the local authority to this terrible scenario and suggest offering support services based on individual needs.

### Supplementary Information

**Additional file1.** STROBE Statement—Checklist of items that should be included in reports of cross-sectional studies.

**Additional file 2: Table S1. **Weighted Descriptive Statistics for Guinea. **Table S2. **Weighted Descriptive Statistics for Kenya. **Table S3. **Weighted Descriptive Statistics for Mali. **Table S4. **Weighted Descriptive Statistics for Nigeria. **Table S5. **Weighted Descriptive Statistics for Senegal.

## Data Availability

All the datasets are freely available for Research in the DHS website. All the codes and materials are available via request to the corresponding author.

## Abbreviations

MIS: Malaria Indicator Survey
LMICs: Low- and middle-income countries
ACT: Artemisinin-based combination therapy
MERG: Monitoring and Evaluation Working Group
DHS: Demographic and Health Surveys
EA: Enumeration Area
AIC: Akaike Information Criterion
VIF: Variance inflation factor
WHO: World Health Organization
