# Supplementary material for: Examining the disparities of anti-malarial drug consumption among children under the age of five: a study of 5 malaria-endemic countries
Source: Malar J. 2023 Dec 5;22:370. doi: 10.1186/s12936-023-04805-x (PMC10696736; doi:10.1186/s12936-023-04805-x)
Supplement: Supplementary file 2 — Additional file 2: Table S1. Weighted Descriptive Statistics for Guinea. Table S2. Weighted Descriptive Statistics for Kenya. Table S3. Weighted Descriptive Statistics for Mali. Table S4. Weighted Descriptive Statistics for Nigeria. Table S5. Weighted Descriptive Statistics for Senegal. [file 12936_2023_4805_MOESM2_ESM.docx]

**Region wise Antimalarial Percentage**

**Supplementary table 1:** Weighted Descriptive Statistics for Guinea.

| Name of the Region | Had Malaria, N (%) | Antimalarial Taken for Malaria, N (%) | Did Not Take Antimalarial for Malaria, N (%) |
| --- | --- | --- | --- |
| Boke | 46.4 (43.6) | 36.1 (77.9) | 10.2 (22.0) |
| Conakry | 37.7 (31.8) | 14.5 (38.5) | 23.2 (61.5) |
| Faranah | 37.1 (47.4) | 26.4 (71.2) | 10.6 (28.7) |
| Kankan | 72.1 (36.4) | 40.9 (56.8) | 31.1 (43.1) |
| Kindia | 47.8 (34.9) | 29.7 (62.2) | 18.0 (37.7) |
| Labe | 19.7 (21.6) | 9.3 (47.5) | 10.3 (52.5) |
| Mamou | 18.2 (39.6) | 15.6 (85.5) | 2.6 (14.4) |
| Nzerekore | 74.2 (58.9) | 61.3 (82.6) | 12.9 (17.3) |

**Supplementary table 2:** Weighted Descriptive Statistics for Kenya

| Name of the Region | Had Malaria, N (%) | Antimalarial Taken for Malaria, N (%) | Did Not Take Antimalarial for Malaria, N (%) |
| --- | --- | --- | --- |
| Coast | 11.1 (15.8) | 1.3 (11.7) | 9.7 (88.2) |
| North Eastern | 0.4 (2.2) | 0 (0) | 0.4 (100) |
| Eastern | 1.7 (7.2) | 0 (0) | 1.7 (100) |
| Central | 0 (0) | - | - |
| Rift Valley | 28.5 (23.4) | 5.4 (19.0) | 23.1 (80.9) |
| Western | 34.2 (33.2) | 2.5 (7.5) | 31.6 (92.5) |
| Nyanza | 51.4 (42.8) | 2.3 (4.6) | 49.0 (95.3) |
| Nairobi | 0 (0) | - | - |

**Supplementary table 3:** Weighted Descriptive Statistics for Mali

| Name of the Region | Had Malaria, N (%) | Antimalarial Taken for Malaria, N (%) | Did Not Take Antimalarial for Malaria, N (%) |
| --- | --- | --- | --- |
| Kayes | 158.9 (32.4) | 96.1 (60.4) | 62.8 (39.5) |
| Koulikoro | 170.3 (45.2) | 142.8 (83.8) | 27.5 (16.2) |
| Sikasso | 188.5 (39.5) | 132.4 (70.2) | 56.1 (29.7) |
| Segou | 202.9 (48.1) | 148.1 (72.9) | 54.8 (27.0) |
| Mopti | 121.7 (38.3) | 74.6 (61.3) | 47.1 (38.6) |
| Tombouctou | 45.6 (50.9) | 27.8 (60.9) | 17.8 (39.0) |
| Gao | 18.1 (39.8) | 14.7 (81.4) | 3.4 (18.5) |
| Kidal | 0.3 (35.4) | 0.07 (21.4) | 0.3 (78.5) |
| Bamako | 53.9 (20.4) | 39.9 (73.9) | 14.1 (26.1) |

**Supplementary table 4:** Weighted Descriptive Statistics for Nigeria

| Name of the Region | Had Malaria, N (%) | Antimalarial Taken for Malaria, N (%) | Did Not Take Antimalarial for Malaria, N (%) |
| --- | --- | --- | --- |
| Sokoto | 125.5 (48.9) | 12.4 (9.9) | 113 (90.1) |
| Zamfara | 53.2 (53.5) | 17.9 (33.6) | 35.3 (66.3) |
| Katsina | 196.3 (67.2) | 8.2 (4.2) | 188.1 (95.8) |
| Jigawa | 101.4 (45.0) | 14.6 (14.4) | 86.7 (85.6) |
| Yobe | 67.2 (41.9) | 22.3 (33.3) | 44.8 (66.7) |
| Borno | 6.8 (13.9) | 0 (0) | 6.9 (100) |
| Adamawa | 20.7 (37.2) | 11.9 (57.4) | 8.8 (42.5) |
| Gombe | 13.0 (35.4) | 0.9 (6.8) | 12.1 (93.2) |
| Bauchi | 171.8 (55.2) | 74.0 (43.1) | 97.7 (56.9) |
| Kano | 50.2 (20.7) | 18.1 (36.0) | 32.1 (64.0) |
| Kaduna | 130.6 (45.6) | 14.2 (10.8) | 116.4 (89.1) |
| Kebbi | 184.9 (50.1) | 1.5 (0.8) | 183.4 (99.2) |
| Niger | 102.7 (49.1) | 22.0 (21.5) | 80.7 (78.5) |
| Federal Capital Territory | 28.3 (56.5) | 21.9 (77.5) | 6.3 (22.4) |
| Nasarawa | 22.6 (57.2) | 17.6 (78.2) | 4.9 (21.8) |
| Plateau | 8.5 (23.1) | 0.5 (5.4) | 8.1 (94.5) |
| Taraba | 30.8 (57.0) | 13.7 (44.5) | 17.1 (55.5) |
| Benue | 30.8 (63.8) | 25.8 (83.8) | 5.0 (16.2) |
| Kogi | 17.6 (41) | 15.1 (85.7) | 2.5 (14.2) |
| Kwara | 22.2 (52.1) | 10.8 (48.9) | 11.3 (51.1) |
| Oyo | 29.4 (55.4) | 19.8 (67.2) | 9.6 (32.7) |
| Osun | 9.4 (38.2) | 6.4 (68.5) | 3.0 (31.4) |
| Ekiti | 1.7 (13.9) | 1.7 (100) | 0 (0) |
| Ondo | 4.9 (36.8) | 2.3 (47.4) | 2.6 (52.6) |
| Edo | 28.6 (60.8) | 14.7 (51.6) | 13.8 (48.4) |
| Anambra | 45.4 (74.1) | 35.6 (78.3) | 9.8 (21.7) |
| Enugu | 16.2 (47.4) | 10.6 (65.2) | 5.6 (34.8) |
| Ebonyi | 36.1 (45.2) | 21.3 (59.1) | 14.7 (40.9) |
| Cross River | 29.3 (47.2) | 14.1 (48.2) | 15.2 (51.8) |
| Akwa Ibom | 13.1 (12.2) | 8.8 (67.1) | 4.3 (32.8) |
| Abia | 38.3 (61.8) | 26.7 (69.7) | 11.6 (30.3) |
| Imo | 16.3 (38.7) | 8.7 (53.6) | 7.6 (46.4) |
| Rivers | 35.3 (54.3) | 12.6 (35.7) | 22.7 (64.2) |
| Bayelsa | 10.7 (43.8) | 3.0 (28.3) | 7.6 (71.7) |
| Delta | 19.5 (19.7) | 12.4 (63.6) | 7.1 (36.3) |
| Lagos | 38.4 (32.1) | 28.9 (75.2) | 9.5 (24.7) |
| Ogun | 17.3 (42.7) | 9.7 (55.8) | 7.6 (44.2) |

**Supplementary table 5:** Weighted Descriptive Statistics for Senegal

| Name of the Region | Had Malaria, N (%) | Antimalarial Taken for Malaria, N (%) | Did Not Take Antimalarial for Malaria, N (%) |
| --- | --- | --- | --- |
| Dakar | 11.5 (4.4) | 0 (0) | 11.5 (100) |
| Ziguinchor | 4.8 (19.0) | 0.7 (14.9) | 4.1 (85.1) |
| Diourbel | 41.1 (12.2) | 13.0 (31.6) | 28.1 (68.4) |
| Saint-Louis | 4.0 (4.4) | 1.3 (34.0) | 2.6 (66.0) |
| Tambacounda | 18.0 (23.1) | 4.8 (27.0) | 13.2 (72.9) |
| Kaolack | 28.2 (28.3) | 0 (0) | 28.2 (100) |
| Thies | 8.5 (6.8) | 0 (0) | 8.5 (100) |
| Louga | 2.9 (2.4) | 0 (0) | 2.9 (100) |
| Fatick | 13.5 (11.6) | 0 (0) | 13.5 (100) |
| Kolda | 13.6 (14.5) | 2.8 (20.9) | 10.7 (79.0) |
| Matam | 14.5 (58.8) | 0.9 (6.2) | 13.6 (93.8) |
| Kaffrine | 37.6 (19.6) | 0 (0) | 37.6 (100) |
| Kedougou | 3.1 (15.3) | 0.4 (13.1) | 2.7 (86.8) |
| Sedhiou | 5.9 (24.4) | 1.8 (30.0) | 4.2 (70.0) |
